# Formation, Transmission, and Dynamic Evolution of a Multidrug-Resistant Chromosomally Integrated Plasmid in *Salmonella* Spp.

**DOI:** 10.3389/fmicb.2022.846954

**Published:** 2022-04-06

**Authors:** Man-Xia Chang, Jing Zhang, Jin-Fei Zhang, Xiao-Min Ding, Yang Lu, Jie Zhang, Ruichao Li, Hong-Xia Jiang

**Affiliations:** ^1^Guangdong Key Laboratory for Veterinary Drug Development and Safety Evaluation, College of Veterinary Medicine, South China Agricultural University, Guangzhou, China; ^2^Guangdong Laboratory for Lingnan Modern Agriculture, Guangzhou, China; ^3^Jiangsu Co-Innovation Center for Prevention and Control of Important Animal Infectious Diseases and Zoonoses, College of Veterinary Medicine, Yangzhou University, Yangzhou, China

**Keywords:** multidrug resistance, cointegrate, homologous recombination, Hfr strain, heterogeneous mobile genetic elements

## Abstract

IncHI2 plasmids, possessing high flexibility and genetic plasticity, play a vital role in the acquisition and transmission of resistance determinants. Polymorphic mobile genetic elements (MGEs) generated by a chromosomally integrated IncHI2 plasmid in an individual *Salmonella* isolate have not yet been detected, and the mechanisms of the formation, excision, and dynamic evolution of a multidrug-resistant chromosomally integrated plasmid (MRCP) have remained obscure. Herein, we identified a 260-kb *bla*_CTX–M–55_-*qnrS1*-bearing IncHI2 plasmid within a *Salmonella* Muenster strain. Plenty of heterogeneous MGEs (new *Escherichia coli* chromosomally integrated plasmid or circular plasmids with different profiles) were yielded when this MRCP was conjugated into *E. coli* J53 with a transfer frequency of 10^–4^–10^–5^ transconjugants per donor. A bioinformatic analysis indicated that replicative transposition and homologous recombination of IS*26* elements were particularly active, and the truncated Tn*1721* also played a vital role in the formation of MRCP offspring. More importantly, when released from the chromosome, MRCP could capture and co-transfer adjacent chromosomal segments to form larger plasmid progeny than itself. Stability and growth kinetics assays showed that the biological characteristics of MRCP progeny were differentiated. This study provides an insight into a flexible existence of MRCP. The conversion between vertical and horizontal transmission endowed MRCP with genetic stability as a chromosomal coding structure and transferability as extra-chromosomal elements. This alternation may accelerate the acquisition and persistence of antibiotic resistance of clinical pathogens and enhance their ability to respond to adverse environments, which poses a great challenge to the traditional antibiotic treatment.

## Introduction

With the increased usage of antibiotics for the control and treatment of infectious diseases, antibiotic resistance (AR) has emerged rapidly at a global scale and spread faster than previously thought. Mobile genetic elements (MGEs) are crucial transmission factors for antibiotic resistance genes (ARGs). Some MGEs (e.g., insertion sequences and transposons) are capable of transferring ARGs to new locations in the same or different DNA molecules within a single cell, while others are capable of delivering ARGs between host cells (e.g., plasmids, integrative conjugative elements, and prophages). Bacterial genomes exhibiting dynamic plasticity could be shaped by horizontal gene transfer (HGT) and genome rearrangement mediated by MGEs (Brown-Jaque et al., 2015; Delavat et al., 2017; Partridge et al., 2018).

Plasmids are important vehicles for the carriage of other MGEs and usually acquire ARGs associated with these elements (Partridge et al., 2018). IncHI2 plasmids are very flexible and genetically plastic, and the highly active traits in acquiring antibiotic resistance determinants make them the primary vector responsible for ARGs transmission (Garcia et al., 2007; Cain and Hall, 2012; Chen et al., 2016; Guo et al., 2016; Wong et al., 2016; Li et al., 2017b,^2020^; Fang et al., 2018; Zhao et al., 2018; Wyrsch et al., 2019; Zingali et al., 2020). Cephalosporin resistance genes *bla*_CTX–M_ have often been found on diverse transmissible plasmids (Canton et al., 2012). In recent years, however, an increasing number of *bla*_CTX–M_ genes have been identified in chromosomes of Enterobacteriaceae (Fabre et al., 2009; Coelho et al., 2010; Hirai et al., 2013; Hamamoto and Hirai, 2019). The transfer mechanism of ARGs from plasmids to chromosomes leads to the long-term persistence of antibiotic-resistant pathogens and imposes concerns on the potential recurrence of diseases. A thorough understanding of the molecular mechanisms underlying the reshuffling of DNA elements may help to establish new strategies to address the problems of AR.

With the bacterial conjugation discovered in the mid-early twentieth century, high-frequency recombination (Hfr) bacteria possessing the entire F factor integrated into the *Escherichia coli* chromosome have been deeply studied at that time. Hfr strains can reform the F factor or the F-prime (F’) factor as a free plasmid independent of the chromosome. The difference between F and F’ factor is that the latter harbors a portion of the donor’s chromosome. Researchers showed that a few R factors in *E. coli* are also capable of transferring chromosomal markers and also form an R’ factor with low frequency (Curtiss, 1969; Arutyunov and Frost, 2013; Villa and Viñas, 2019). The authors suggested that, with the exception of the reported Hfr donors, all other conjugal fertility factors should be referred to as plasmids until a chromosomal integration state can be demonstrated (Curtiss, 1969).

Here, we report the isolation of a multidrug-resistant chromosomally integrated plasmid (MRCP) belonging to the IncHI2 family from a *Salmonella* strain. According to the definition of Hfr strains, PJM1 can be defined as a SHfr_HI2_^R^ strain. MRCP possesses the ability of generating heterogeneous forms as well as capturing and co-transferring adjacent chromosomal segments. More importantly, the SHfr_HI2_^R^ demonstrated the capability to transmit vertically in a host-integrated state or horizontally through excision from the chromosome. This biphasic lifestyle confers MRCP genetic stability and transferability and greatly expands the range of possibilities of AR to be transmitted.

## Materials and Methods

### Bacterial Strains and Antimicrobial Susceptibility Testing

All bacterial strains used in this study are listed in Table 1. AR phenotypes of these strains and their transconjugants against cephalosporin and ciprofloxacin (Table 1) were determined using the agar dilution method following the guidelines of the Clinical and Laboratory Standards Institute (CLSI, 2019).

**TABLE 1:** Characteristics of *bla*_CTX–M–55_-bearing positive strains and their corresponding transconjugants.

Role in the conjugation assays	Strain name	*bla*_CTX–M–55_ location	MIC (mg/l)		Description
			CTX	CIP	
Donor strains in the first-round conjugation	PJM1	Chromosome	256	0.25	Clinical *Salmonella* strains co-resistant to cephalosporin and ciprofloxacin
	OYZ4	258-kb plasmid	256	0.25	

Recipient strain in the first-round conjugation	J53	NA*^c^*	0.008	0.015	*E. coli* model strain resistant to sodium azide

Recipient strain in the second-round conjugation	SL1344	NA	0.03	0.008	Model strains of *S. typhimurium*
	str.14028s		0.06	0.015	
	M39	NA	0.06	0.06	Clinical strain *S.* Derby

J53-transconjugants in the first-round conjugation	C-OYZ4	258-kb plasmid	64	0.25	Transconjugants of recipient strain *E. coli* J53 and donor strain OYZ4

	PJ-T0	236-kb plasmid	64	0.25	
	PJ-T1*^b^*	452-kb plasmid	32	0.25	Transconjugants of recipient strain *E. coli* J53 (the characters “PJ” for the donor strain PJM1, “T” for “transconjugants,” and the numbers for the order of transconjugants obtained)
J53-transconjugants in the first-round conjugation, as well as donor strains in the second-round conjugation	PJ-T13*^a^*	Chromosome	128	0.5	
	PJ-T7	250-kb plasmid	64	0.5	
	PJ-T15*^b^*	320-kb plasmid	32	0.25	
	PJ-T16	250-kb plasmid	64	0.5	
	PJ-T17*^b^*	213-kb plasmid	64	0.5	

	PJ-T0-SL	236-kb plasmid	256	0.25	
	PJ-T1-SL	452-kb plasmid	256	0.5	
	PJ-T7-SL	250-kb plasmid	256	0.25	
	PJ-T15-SL	320-kb plasmid	256	0.25	
	PJ-T16-SL	250-kb plasmid	256	0.25	Transconjugants of recipient strain *Salmonella* [“SL,” “LT,” or “M” stand for recipient strains SL1344, str.14028s, or M39, and the remainder represent the donor strains in second-round conjugation (e.g., PJ-T0-SL refers to the transconjugant of donor PJ-T0 and recipient SL1344)]
	PJ-T17-SL	213-kb plasmid	256	0.25	
Transconjugants in the second-round conjugation	PJ-T0-LT	236-kb plasmid	256	0.25	
	PJ-T1-LT	452-kb plasmid	256	0.25	
	PJ-T7-LT	250-kb plasmid	256	0.25	
	PJ-T15-LT	320-kb plasmid	256	0.25	
	PJ-T16-LT	250-kb plasmid	256	0.25	
	PJ-T17-LT	213-kb plasmid	256	0.25	
	PJ-T0-M	236-kb plasmid	256	1	
	PJ-T7-M	250-kb plasmid	256	1	
	PJ-T16-M	250-kb plasmid	256	1	

*^a^ PJ-T13 failed to transfer ARGs to SL1344, str.14028s, and M39.*

*^b^ PJ-T1, PJ-T15, and PJ-T17 could transfer ARGs to SL1344 and str.14028s but failed to transfer to M39.*

*^c^ NA, not applicable.*

### WGS, Bioinformatic Analysis, and PCR

Complete genome sequences were annotated using the RAST server (Overbeek et al., 2014). Tools including EasyFig (Sullivan et al., 2011), BRIG (Alikhan et al., 2011), and BWA-MEM (Li et al., 2018) were used for bioinformatic analysis. Primers for PCR are listed in Supplementary Table 1.

### Mating Experiments, S1-PFGE, and Southern Hybridization

The transferability of MRCP and its representative progeny were investigated by filter mating. The donor and recipient strains of the first-round conjugation assay were SHfr_HI2_^R^ strain PJM1 and *E. coli* J53, respectively. The J53-transconjugants obtained in the first-round conjugation were used as the donor strains of the second-round conjugation and *Salmonella* strains (SL1344, ATCC 14028, and M39) as recipient strains. Briefly, when grown to log phase in Luria-Bertani (LB) broth, donor and recipient cultures were mixed in a 1:1 ratio; filtered through 0.22-μm pore size membranes (HuanKai Microbial, Guangzhou, China) placed on pre-warmed LB agar plates; and incubated for 16 h at 30, 37, and 42°C, respectively. For *E. coli* J53, putative J53-transconjugants were selected from MacConkey agar plates supplemented with cefotaxime (4 mg/l) (Sigma-Aldrich, Guangzhou, China) plus sodium azide (100 mg/l) (Sigma-Aldrich, Guangzhou, China), and for *Salmonella* SL1344, ATCC 14028, and M39, the second-round transconjugants were selected on xylose lysine desoxycholate (XLD) agar (HuanKai Microbial, Guangzhou, China) plates supplemented with cefotaxime (2 μg/ml). Transconjugants were reconfirmed by *Xba*I-PFGE (*Xba*I enzyme, Takara Bio Inc., Beijing, China) and PCR screening for *bla*_CTX–M–55_ and *rep*HI2. Transfer frequencies were calculated as the number of transconjugants obtained per recipient. S1-PFGE (S1 enzyme, Takara Bio Inc., Beijing, China) followed by southern blot hybridization with digoxigenin-dUTP-labeled probes of *bla*_CTX–M–55_ and *rep*HI2 genes were conducted to obtain the localizations and/or profiles of the integrated/free plasmids (Piekarowicz et al., 1968; Yang et al., 2017; Zhang et al., 2019).

### Stability and Growth Kinetics Assays

The stability of MRCP in strain PJM1 and its progeny in *E. coli* J53 were studied by continuous passage for 30 days. Specifically, 5 μl of bacteria culture per strain were diluted in 5 ml of antibiotic-free fresh LB broth (1:1,000) every 24 h and incubated at 30°C and 180 rpm. Three independent experiments were conducted in triplicate under each condition. The serially diluted cultures of PJM1 and its J53-transconjugants grown to 20th and 30th days were plated on XLD and MacConkey agar plates, respectively. Fifty colonies were selected, and the presence of cephalosporin resistance phenotypes and HI2 replicon was confirmed by PCR. Plasmid stability was calculated as the proportion of cells harboring both *bla*_CTX–M–55_ and *rep*HI2 in the 50 selected colonies. The growth kinetics of PJM1, OYZ4, *E. coli* J53, and the J53-transconjugants (PJ-T0, PJ-T7, PJ-T13, PJ-T16, PJ-T17, and C-OYZ4) were studied following the method reported in literature (Lv et al., 2020). The maximum growth rate (μ_*max*_), maximum OD_600_ (OD_*max*_), the area under the growth curve (AUC), and the duration of the lag phase (lag) were calculated to evaluate the growth ability of the J53-transconjugants.

### Data Availability

The complete sequences of the plasmids and the chromosomes have been deposited in GenBank under the accession numbers pOYZ4 (MN539018), pPJ-T0-236kb (MN539017), pPJ-T7-250kb (OL598600), pPJ-T16-250kb (OL598601), pPJ-T17-213kb (OL598602), PJ-T1-452kb (OM317741), PJ-T15-320kb (OM317740), PJ-T13-chr (CP087110), and PJM1-chr (CP045038).

## Results

### The Discovery of Heterogeneous Forms Yielded by a Multidrug-Resistant Chromosomally Integrated Plasmid in PJM1 by Conjugation Assays

A 236-kb IncHI2 plasmid, which carried chromosomally located *bla*_CTX–M–55_ and *qnrS1* in *Salmonella* Muenster PJM1, was detected in the corresponding J53-transconjugant C-PJM1 (renamed PJ-T0 in this study) as previously described (Zhang et al., 2019). The unique HI2 replicon located in the PJM1 chromosome was subsequently confirmed by southern hybridization with *rep*HI2 probe (Supplementary Figure 1). Interestingly, when we repeated the conjugation experiment, the size of the plasmid transferred to *E. coli* J53 was variable, but remained unchanged when re-transferred to *Salmonella* strains (Table 1). In contrast, another circular IncHI2 plasmid in *S.* Chester OYZ4 (Zhang et al., 2019) remained constant when transferred to *E. coli* J53 and other receptor strains by conjugation. Considering the facts above, we suspected that there was a multidrug-resistant plasmid (MRCP) integrated in PJM1 chromosome, resulting in the generation of a SHfr_HI2_^R^ strain.

To verify the characteristics of this SHfr_HI2_^R^ strain, 95 and 90 *E. coli* J53-transconjugants were randomly selected *via* conjugation at 30 and 37°C, respectively. The cephalosporin and fluoroquinolone co-resistance phenotype, as well as *bla*_CTX–M–55_, *qnrS1*, and *rep*HI2 genes, was detected in all J53-transconjugants. *Xba*I-PFGE reconfirmed that the transconjugants were indeed *E. coli* J53. S1-PFGE profiles of the 185 J53-transconjugants showed that the MRCP in PJM1 was transferred to *E. coli* J53, yielding a wide range of plasmid sizes (187–459-kb) at 30 and 37°C (Supplementary Figure 2).

Three clusters including 14 subtypes were obtained by clustering analysis on the S1-PFGE profiles (Supplementary Figure 3A). Considering the size error margin of S1-PFGE prediction, plasmids showing a similar size (± 20-kb) were regarded as belonging to the same subtype. Cluster B (circular plasmid, sizes < 260-kb) had the highest yields, while cluster A (circular factors, size > 260-kb) were more frequently detected after conjugation at 30°C. Cluster C (the re-formation of plasmid-chromosomal cointegrates in *E. coli* J53) only seemed to occur at a very low rate at 37°C (Supplementary Figure 3A). Moreover, *siiF* was detected on pPJ-T1-452kb and pPJ-T15-320kb of cluster A by southern hybridization (Supplementary Figure 3B). Interestingly, the *siiABCDEF* gene cluster is present in *Salmonella* sp. but not in *E. coli* (Morgan et al., 2004; Barlag and Hensel, 2015). These results prompted us to trace why PJM1 has the capability to yield such a wide range of plasmid sizes and how *siiF* changed from being exclusive to *Salmonella* to being shared by *E. coli* and *Salmonella*.

### Genetic Characteristics of Multidrug-Resistant Chromosomally Integrated Plasmid and Its Differentiated Descendants

**FIGURE 1:**
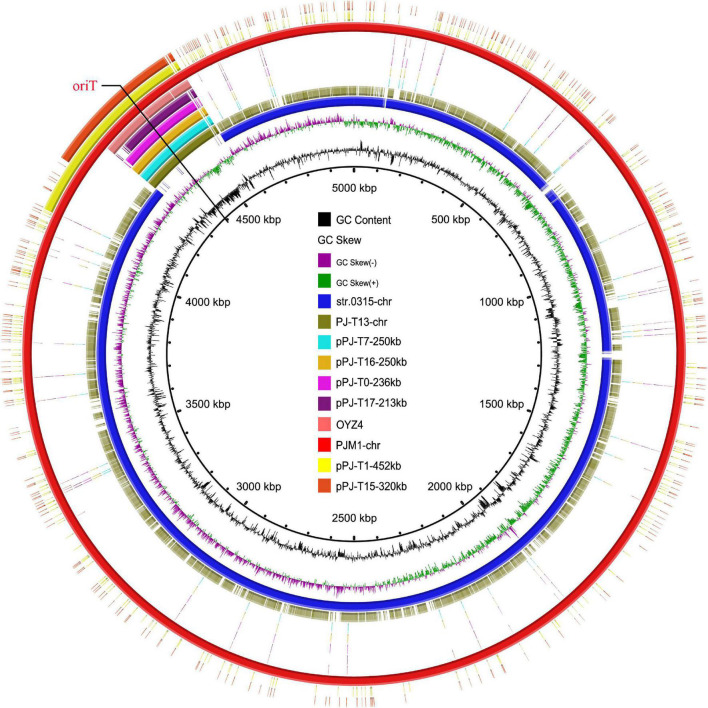
Circular alignment of chromosomes PJM1-chr, str.0315-chr, and PJ-T13-chr and plasmids pPJ-T17-213kb, pPJ-T0-236kb, pPJ-T16-250kb, pPJ-T7-250kb, pOYZ4, pPJ-T1-452kb, and pPJ-T15-320kb. The red circle denotes chromosome PJM1-chr as a reference. Bright blue and olive-green circles represent str.0315-chr (NZ_CP019198) and PJ-T13-chr, respectively; aqua, sallow, violet, deep purple, coral red, yellow, and orange-red circles depict the plasmids pPJ-T7-250kb, pPJ-T16-250kb, pPJ-T0-236kb, pPJ-T17-213kb, pOYZ4, pPJ-T1-452kb, and pPJ-T15-320kb, respectively.

To investigate the formation mechanism of the MRCP heterogeneous progeny, the accurate genome structures of parental strain PJM1 and its representative progeny (PJ-T13, plasmid integrated into *E. coli* J53 chromosome; pPJ-T0-230kb, pPJ-T16-250kb, pPJ-T17-213kb, pPJ-T1-452kb, and pPJ-T15-320kb, element as a 230-, 250-, 213-, 452-, and 320-kb circular molecule in J53, respectively) were obtained using deep DNA sequencing. The PJM1 chromosome was 5,000,356-bp in length and composed of 4,725 predicted coding sequences (CDSs) with 51.9% GC content. BLASTN analysis indicated that this chromosome exhibited high homology (94.4% coverage and 99.9% identity) to *S.* Muenster str. 0315 (NZ_CP019198) isolated from a fecal sample from a dairy farm in the United States. Comparison with the chromosome of str. 0315 showed that the PJM1 chromosome featured an extra DNA fragment, namely, MRCP (Figure 1). MRCP was 261,307-bp with 46.7% GC and 306 CDSs and displayed 99% identity with 78.5 and 88.2% coverage to the IncHI2 plasmids pDGSE139 (KM198330) and pCFSA1096 (CP033347), respectively (Supplementary Figure 4).

**FIGURE 2:**
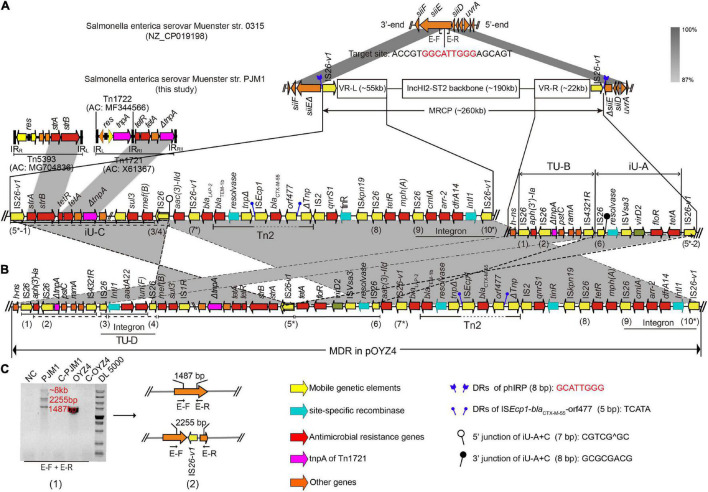
The structure and location of MRCP in PJM1 chromosome. **(A)** Comparison between the genetic structure of PJM1 chromosome and *S*. Muenster str. 0315. **(B)** The left and right variable regions (VR-L and VR-R) of MRCP and the corresponding MDR regions in pOYZ4. The IS*26* elements (IS*26* and IS*26-*v1) in pOYZ4 and MRCP are marked with numbers. **(C)** Gel electrophoretic diagram (1) of PCR amplicons of primer pairs E-F and E-R and the corresponding structure (2) detected. All amplicons were confirmed by sequencing. Primers are listed in Supplementary Table 1.

**FIGURE 3:**
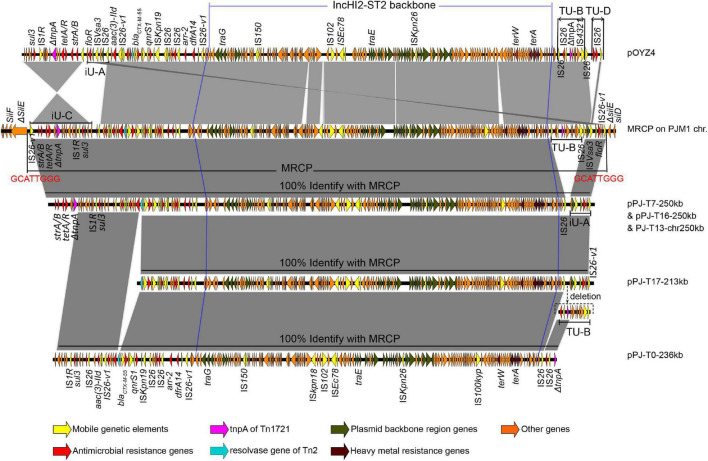
Sequence alignment of the multidrug-resistant chromosomally integrated plasmid (MRCP), its progeny, and pOYZ4. The highlighted GCATTGGG nucleotides represent the duplicated DRs that flanked MRCP. Regions of > 99% identity are marked by gray shading. The positions of the genetic structures iU-A, TU-B, iU-C, and TU-D were marked. iU, inversion unit; TU, translocatable unit.

Interestingly, *S.* Chester OYZ4 obtained from poultry in Guangdong Province, China, at the same time as PJM1 in 2017 carried the circular plasmid pOYZ4, which harbored 327 predicted CDSs distributed among 257,945-bp with a 47% GC content. Importantly, although the two strains belonged to different serotypes and *Xba*I-PFGE profiles (Zhang et al., 2019), pOYZ4 shared a surprisingly high degree of homology (88.46% coverage; 99.71% identity) with MRCP (Supplementary Figure 4 and Figure 2). Similar to pOYZ4, MRCP also possessed an intact IncHI2-ST2 backbone (190-kb), which contained genes for replication (*rep*HIA and *rep*HI2), partition (*par*AB and *par*MR), conjugation (*tra*, *trh*, and *htd*), complete or partial toxin–antitoxin systems (*hipA/hipB* and *higB*), an SOS mutagenesis correlative operon (*umuCD*), and two tellurite resistance operons (*terY3Y2XY1W* and *terZABCDEF)* (Figure 3 and Supplementary Figure 4). Interestingly, the backbone was flanked by two variable regions (VRs) containing multi-resistance genes and four genes encoding site-specific recombinase (Figure 2).

The left VR (VR-L, 55-kb) clustered numerous mosaic genetic elements. The resistance gene cassettes (*cmlA*, *arr2*, and *dfrA14*) integrated into a class 1 integron were at the end of VR-L adjacent to the plasmid backbone, followed by *mph*(A), *tetR*, *qnrS1*, and a 2,971-bp transposition unit (IS*Ecp1*-*bla*_CTX–M–55_-*orf477*), which truncated a complete composite transposon Tn*2*. A partial Tn*5393* variant was detected at the other end of VR-L, which consisted of its left terminal inverted repeats (IR_*L*5393_, 81-bp) and carrying the genes *strA/B*. Importantly, the sequence adjacent to ΔTn*5393* seemed to be the residual variant of Tn*1721* after losing its basic transposon Tn*1722* (ΔTnp1721, 1,789-bp), tetracycline resistance genes, and two 38-bp right terminal repeat variants (IR_*RI*_ and IR_*RII*_) at both ends (Figure 2). The right VR (VR-R, 22-kb) contained four IS*26* elements, a resolvase gene, and many ARGs including *aph (3′)-Ia*, *floR*, and *tet*(A) (Figure 2). Interestingly, a truncated Tn*1721* transposase gene (ΔTnp*1721*, 848-bp) that seemed to be a part of ΔTn*1721* in VR-L was also present in VR-R (Figure 2).

The circular plasmid pPJ-T0-230kb in J53-transconjugant PJ-T0 was 236,068-bp with 46% GC and 311 predicted CDSs (Figures 1, 3 and Supplementary Figure 4). Similarly, the pPJ-T16-250kb in PJ-T16 was 247,473-bp with 47% GC and 298 CDSs, and the pPJ-T17-213kb in PJ-T17 was 213,369-bp with 46% GC and 247 CDSs. All the plasmids showed a high homology with the MRCP (100% identity with 95.17, 97.35, and 90.83% coverage, respectively), and they all shared the same IncHI2-ST2 backbone as expected (Figures 1, 3 and Supplementary Figure 4). Interestingly, the fragment PJ-T13-chr250kb inserted into the J53 chromosomal gene *bglH* was the same as pPJ-T16-250kb (Figures 1, 3 and Supplementary Figure 4). That is, the SHfr_HI2_^R^ strain could not only form free IncHI2 plasmids but also directly integrate into the chromosome of the recipient, giving place to a new MRCP in *E. coli*. On the other hand, pPJ-T1-452kb in PJ-T1 was 452,929-bp with 49% GC and 464 CDSs, and pPJ-T15-320kb in PJ-T15 was 331,203-bp with 48% GC and 362 CDSs. Apart from containing the 250-kb MRCP, both pPJ-T1-452kb and pPJ-T15-320kb harbored a segment of PJM1 chromosome (Figure 1), which demonstrated the capability of MRCP to capture and co-transfer adjacent chromosomal segments in PJM1. Therefore, the circular factors (pPJ-T1-452kb and pPJ-T15-320kb) in cluster B corresponded to R_HI2_’ variants that consisted of MRCP and a portion of a chromosomal fragment.

### Dynamic Evolution and Prevalence of Multidrug-Resistant Chromosomally Integrated Plasmid

To verify the dynamic evolution of MRCP during bacterial division, primers E-F and E-R from the 3’-end and 5’-end of *siiE* were used to amplify the probable residual regions after the excision of diverse MRCP descendants in the evolutionary PJM1 population (Figure 2A). Three products (1.5, 2.3, and > 5-kb) were obtained in PJM1. DNA sequencing showed that the 1.5-kb product, which was the same as the product in OYZ4, aligned with *siiE* exactly. The 2.3-kb product consisted of the truncated *siiE* and a IS*26-v1* that interrupted *siiE* (Figure 2C). Despite many attempts, we still could not obtain the complete sequence of product > 5-kb by Sanger sequencing, which seemed to be a mixture of different sequences with the same adjacent sequence. This phenomenon was consistent with a diversity of J53-transconjugants obtained in the conjugation assays, indicating that the MRCP in the chromosomes of the PJM1 population were in dynamic equilibrium during bacterial division. If MRCP is deleted from the PJM1 chromosome, some sequence traces would be left behind (e.g., if MRCP is deleted in the form of pPJ-T7, it leaves a trace *siiE*Δ-IS*26-v1*-Δ*siiE* in the PJM1 chromosome; if MRCP is deleted as pPJ-T7, it leaves *siiE*Δ-IS*26-v1-strA/B-tetA/R*-ΔTn*1721-*IS*Vsa3-floR*-Δ*siiE*; and in the case of pPJT17, *siiE*Δ-IS*26-v1-strA/B-tetA/R*-ΔTn*1721-*IS*1R-sul3-*IS*26-aac(3)-IId*- IS*26-v1-Tn2*Δ-Δ*siiE* would be left, etc.). Due to the heterogeneous forms generated by MRCP, the sequence traces left changed with different deletions. Accordingly, quantitative traces (i.e., decayed MRCP) should theoretically be left in the PJM1 subpopulation. To further verify the dynamic evolution mechanisms of MRCP, the raw data of PJM1 nanopore (ONT) long-reads have also been reanalyzed, but no reads covering the decayed MRCP were found.

### The Molecular Mechanisms of Formation and Dynamic Transmission of Multidrug-Resistant Chromosomally Integrated Plasmid

**FIGURE 4:**
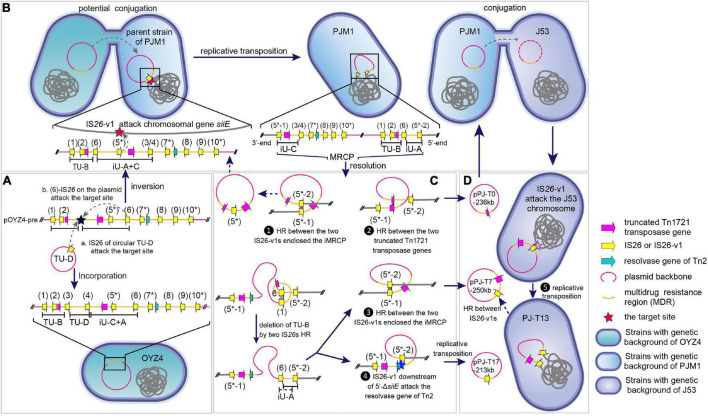
Presumptive formation and multiple transmission form of MRCP. **(A)** a. TU-D was inserted into the precursor plasmid pOYZ4-pre *via* conservative transposition, forming pOYZ4. b. The IS*26* copy 6 in pOYZ4-pre attacked the target site (black star) in the opposite DNA strand, causing reversal of iU-C + A segment. **(B)** IS*26-v1* in iU-A + C segment attacked the chromosomal *siiE* of PJM1 during conjugation, forming the cointegrate by intermolecular replicative transposition. The plasmid in PJM1 chromosome was renamed MRCP according to its characteristics. **(C)** Diverse resolution of MRCP. ❶ The direct homologous recombination between IS*26-v1* copies 5*-1 and 5*-2 mediated the generation of a plasmid identical to pPOYZ4-pre except for reversed iU-C + A. ❷ The direct homologous recombination between two truncated Tn*1721* led to plasmid pPJ-T0-236kb. ❸ Deletion of TU-B and homologous recombination between IS*26-v1* copies 5*-1 and 5*-2 yielded plasmid pPJ-T7-250kb or pPJ-T16-250kb. ❹ Intramolecular replicative transposition resulted in the formation of plasmid pPJ-T17-213kb. After TU-B being deleted by homologous recombination, IS*26-v1* copy 5*-2 attacked the target site (blue star) in the resolvase gene of Tn*2*, forming plasmid pPJ-T17-213kb. **(D)** ❺ Intermolecular replicative transposition led to PJ-T13-chr250kb. When a plasmid such as pPJ-T7-250kb was transferred into *E. coli* J53 by conjugation, IS*26-v1* copy 5 attacked J53 chromosome, and PJ-T13-chr250kb in PJ-T13 was formed.

In the two VRs of MRCP, 10 copies of IS*26* elements (IS*26* or IS*26* variant, IS*26*-v1) were identified (Supplementary Table 2), corresponding to the 10 copies in pOYZ4 (Figures 2B, 4). Almost all IS*26*s copies were intact, except that copy 8 in MRCP and pOYZ4 lacked the right terminal inverted repeat (IR_*R*_), and copy 2 (present in MRCP) featured a single-base deletion (T) at position + 376, which resulted in a frameshift mutation (Figures 2B, 4). None of the IS*26*s were flanked by target site duplications (TSDs) that are typical for insertion sequences of this family. The 8-bp sequence 5’-CGTCGCGC-3’ (Figure 2B, black lollipop) downstream of IS*26* copy 6 and the 7-bp incomplete reverse complement sequence 5’- GC^CGACG-3’ (Figure 2B, white lollipop) upstream of IS*26* copy 3/4 of MRCP (“copy 3/4” represents IS*26* in MRCP and corresponds with the IS*26* copy 3 or IS*26* copy 4 in pOYZ4) provided evidence for inverse intramolecular replicative transposition, which reversed the 40-kb segment-designated iU-A + C (Figures 2B, 4A). The IS*26-*v1 copy 5* (an asterisk was used to distinguish IS*26-v1* from IS*26*) in iU-A + C interacted with the target site (GCATTGGG) in chromosomal *siiE*, resulting in the integration of the entire plasmid into the PJM1 chromosome to form the MRCP and the splitting of iU-A + C into iU-A and iU-C. Meanwhile, IS*26-*v1 copies 5*-1 and 5*-2 at each junction between MRCP and the PJM1 chromosome were generated by direct replication of IS*26-*v1 copy 5* (Figure 4B).

As recently published, when a central fragment is flanked by two identical IS*26*s, this fragment with one adjacent IS constitutes a translocatable unit (TU), which results in the excision and movement of ARGs between different replicons (from one plasmid to another or to the chromosome, and vice versa) (Harmer et al., 2020). Accordingly, the inversion of iU-A + C also led to the formation of TU-B [IS*26*-*aph (3′)-Ia*-IS*26-*ΔTn*1721*-IS*4321R*] in MRCP and in the same orientation of ΔTn*1721* within TU-B and iU-C (Figure 4B). A single homologous recombination (HR) event between two ΔTn*1721* released pPJ-T0-236kb (Figure 4C). Besides, the HR between two IS*26*s (copies 1 and 6) mediated the deletion of TU-B also carrying ΔTn*1721*. Subsequently, two parallel events may have taken place on the chromosome lacking TU-B: (i) The HR between IS*26-*v1 copies 5*-1 and 5*-2 released pPJ-T7-250kb, resulting in the chromosome modified by the IS*26-v1* flanked by the TSD. (ii) IS*26-*v1 copy 5*-2 downstream of 5′-Δ*siiE* interacted with the gene encoding Tn*2* resolvase on the same DNA strand giving place to pPJ-T17-213kb (Figure 4C). The IS*26-*v1 copy 5* on pPJ-T7-250kb, on the other hand, interacted with the *bgIH* gene on the *E. coli* J53 chromosome and generated the PJ-T13 cointegrate through intermolecular replicative transposition (Figure 4D). Conversely, the HR between IS*26-*v1 copies 5*-1 and 5*-2 converted chromosomal fragment PJ-T13-chr250kb into the circular plasmid pPJ-T7-250kb (Figure 4D).

### Transferability, Stability, and/or Growth Kinetics of Multidrug-Resistant Chromosomally Integrated Plasmid and Its Progeny

**FIGURE 5:**
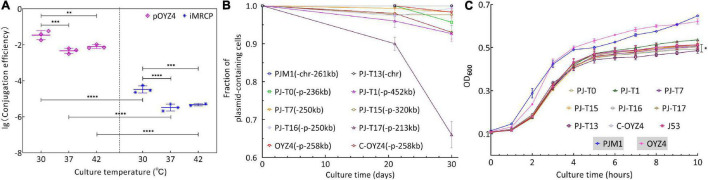
**(A)** Comparison of transfer efficiencies of MRCP and IncHI2 plasmid pOYZ4 into *E. coli* J53. Transfer efficiency (mean ± standard deviation) is calculated based on colony counts of the transconjugant and recipient cells in triplicate. **(B)** Stability of MRCP in PJM1 and its progeny in *E. coli* J53 for 30 days. **(C)** Growth curves of representative J53-transconjugants, recipient strain *E. coli* J53, and donor strain PJM1. *Salmonella* OYZ4 serves here as a contrast strain for PJM1. Considering the interspecific difference, comparisons were performed between the J53-transconjugant and *E. coli* J53, and between PJM1 and OYZ4, respectively. All experiments were repeated independently in triplicate. The error bars denote the standard deviations (*n* = 3). Two-way analysis of variance (ANOVA) with Tukey’s multiple comparison was used to evaluate statistical significance. Significantly different at *P ≤ 0.05; ^**^P ≤ 0.01; ^***^P ≤ 0.001; ^****^P ≤ 0.00001.

To determine the transferability of the SHfr_HI2_^R^ strain PJM1, conjugation experiments were performed at 30, 37, and/or 42°C. Plasmid pOYZ4 was used as a control and *E. coli* J53 as recipient. Compared with the other two culture temperatures, both MRCP and pOYZ4 had the highest transfer frequency at 30°C (Figure 5A). However, the transfer frequency of MRCP (10^–4^–10^–5^ transconjugants per donor) was always significantly lower than that of pOYZ4 (10^–2^–10^–3^ transconjugants per donor) at selected temperatures (*p* < 0.0001) (Figure 5A). Interestingly, after 30 days of passage without antibiotics, MRCP and pOYZ4 were stably maintained in parental strain PJM1 and OYZ4, respectively (Figure 5B). Besides, there was no significant difference in the maximum growth rate (μ_*max*_, *p* > 0.05) and AUC (*p* > 0.05) between PJM1 and OYZ4, but the duration of the lag phase and maximum OD_600_ of PJM1 was significantly higher than that of OYZ4 (lag, *p* < 0.01 and OD_*max*_, *p* < 0.001).

After 30 days of passage, pPJ-T1-452kb, pPJ-T15-320kb, pPJ-T7-250kb, pPJ-T16-250kb, pPJ-T0-236kb, and the chromosomal PJ-T13-chr250kb were stably maintained (plasmid-containing populations fraction > 90%) in their host strains (*E. coli* J53-transconjugants PJ-T1, pPJ-T15, PJ-T7, PJ-T16, and PJ-T0), whereas the stability of plasmid pPJ-T17-213kb was 66% maintained in its host strain PJ-T17 (Figure 5B). As for the growth ability, although there were no significant differences in the maximum growth rate (μ_*max*_, *p* > 0.05) between J53-transconjugants (PJ-T0, PJ-T7, PJ-T13, PJ-T16, PJ-T17, and C-OYZ4) and *E. coli* J53, there were significant differences between PJ-T13 and *E. coli* J53 in the duration of the lag phase (lag, *p* < 0.05), AUC (*p* < 0.05), and maximum OD_600_ (OD_*max*_, *p* < 0.001), implying no significant differences between the other J53-transconjugants and *E. coli* J53 during 10-h assessment, except for a significant difference between PJ-T13 and *E. coli* J53 (Figure 5C). To test the transferability of different progenies of MRCP to other host strains, second-round conjugation assays were performed at 37°C with the representative J53-transconjugants as the donor strains and three *Salmonella* strains (SL1344, str.14028s, and M39) as the recipient strains (Table 1). Plasmids pPJ-T7-250kb, pPJ-T16-250kb, and pPJ-T0-236kb in J53-transconjugants were transferred to the aforementioned *Salmonella* strains; pPJ-T1-452kb, pPJ-T15-320kb, and pPJ-T17-213kb were transferred to SL1344 and str.14028s, but not M39, while chromosomal MGE PJ-T13-chr250kb could not be transferred to any of the three.

## Discussion

Conjugative plasmids are the most important drivers of the evolution of antibiotic-resistant bacteria (Partridge et al., 2018). IncHI2 plasmids are known to be significant vectors of clinically important ARGs encoding resistance to carbapenems (Carattoli, 2013; Zhang et al., 2019), colistin (Li et al., 2017b,^2020^), and quinolones (Carattoli, 2013; Wong et al., 2016). The high flexibility and genetic plasticity of IncHI2 plasmids can confer their hosts a great capability of acquiring ARGs (Garcia et al., 2007; Guo et al., 2016; Li et al., 2017b,^2020^; Fang et al., 2018; Zhao et al., 2018; Wyrsch et al., 2019; Zingali et al., 2020) or to form cointegrates with other plasmids (Garcia et al., 2007; Schluter et al., 2014; Li et al., 2017a; Wong et al., 2017; Fang et al., 2018; Wang et al., 2018; Xie et al., 2018; Chen et al., 2019). In this study, we identified a multidrug-resistant chromosomally integrated plasmid that produced many heterogeneous MGEs during the conjugation process, and the strain PJM1 was defined as SHfr_HI2_^R^ strains according to the definition of Hfr strains.

Segregational instability is the term used to describe the loss of plasmids that occurs during cell division, regardless of whether plasmid carriage is costly or not (Carroll and Wong, 2018). The formation of SHfr_HI2_^R^ strain may be a strategy employed by bacteria to avoid segregational loss while maintaining mobility of the resistance factors contained therein. Thus, the MRCP can be stably inherited in a host-integrated state and spread horizontally by excision from chromosome. On the other hand, although the available experimental methods cannot eliminate duplicate strains in *E. coli* J53-transconjugants harboring heterogeneous MGEs and the errors in plasmid size estimates that may arise after S1-PFGE assays, we confirmed the presence of two different sizes of plasmids in one subgroup by deep DNA sequencing (pPT-T0-230kb and pPT-T17-213kb were clustered in the 211–230-kb subgroup), indicating that MRCP progeny size indeed covered a broad range far beyond our expectations. Besides, we speculated that the temperature may not affect the size range of MRCP progeny, but it would affect the distribution of specific plasmids. In other words, the excision, transmission, and re-integration of MRCP (forming clusters A, B, and C) may be mediated by different enzyme systems, and their corresponding activities may be affected by temperature. The re-formation rate (1.6%) of plasmid-chromosomal cointegrates in *E. coli* J53 is very low and seems to be temperature dependent, consistent with the view of Carroll and Wong (2018) that host chromosome integration is considered an exception because it rarely occurs in the laboratory. More importantly, the capability of MRCP to capture and co-transfer adjacent chromosomal segments was unexpected. Some studies indicated that site-specific recombinase might be responsible for the shuffling of large DNA fragments (Ravatn et al., 1998; Groth and Calos, 2004; Miyazaki and van der Meer, 2013; Lv et al., 2020). However, it seems that the transposase encoded by IS*26-v1* in MRCP, rather than the site-specific recombinases, mediated the conjugative mobilization of adjacent chromosomal segments in *cis*. To the best of our knowledge, IS*26*-mediated plasmid capture of chromosomal fragments has not been reported in *Salmonella*, and the translocation mechanisms of PJM1 chromosomal DNA fragments remain to be further investigated.

No plasmid derivatives containing two ΔTn*1721* were detected, indicating that the deletion of a ΔTn*1721* copy seemed to be an essential prerequisite. HR between two partially identical ΔTn*1721* appeared to be the most straightforward way to resolve MRCP from the PJM1 chromosome. Moreover, IS*26-v1* HR and IS*26-v1-*mediated reactions resulted in the formation or polymorphic resolution of the cointegrate. Meanwhile, we found that IS*26-*mediated inverse intramolecular replicative transposition and TU-B deletion were also involved. We speculate that different IS*26* variants tend to participate in different pathways. In this regard, IS*26-v1* tends to form and resolve the cointegrate between different replicons, whereas IS*26* tends to cause structural variations within a single replicon. An *in vitro* study supports this view that the IS*26* variant with G184N substitution (such as IS*26-v1*) increased Tnp26 activity and the cointegration frequency (Pong et al., 2019).

In the present study, MRCP was autonomously transferred to other organisms at a much lower frequency than pOYZ4. Moreover, the transfer frequency of MRCP was possibly the final result of multi-type excision, conjugation, and plasmid-chromosomal transposition. Additionally, the significantly lower growth ability of PJ-T13 than that of *E. coli* J53 indicated that the initial fitness cost of MRCP progeny increased in a cointegrated state, which is in agreement with the statement that any plasmid integrating into and interrupting important host genes can increase fitness costs (Carroll and Wong, 2018). However, whether the plasmid existed independently in OYZ4 or integrated into PJM1 chromosome, the growth ability of bacteria appeared to be unaffected, suggesting that cointegrate will not pose significant fitness cost to PJM1. This seems to be a paradox, but we rationally believe that the cointegrate is probably species specific or requires a longer evolutionary time to adapt to its bacterial host (Wein et al., 2019). Also, as Carroll and Wong (2018) stated, the genetic background of the host might be an important determinant of plasmid fitness. Namely, a single plasmid beneficial in a strain with a certain genetic background could decrease fitness in the other. Furthermore, we consider that the balance of maintaining the fitness of the host and MRCP offspring might govern the transfer types and transfer frequencies (Delavat et al., 2017). On the other hand, despite the growth disadvantages of PJ-T13, its cointegrate could be maintained stably just like MRCP in PJM1. However, a large proportion of the terminal bacterial population (in the stability assays) had lost pPJ-T17-213kb. The divergence of the polymorphic MGEs indicated that the MRCP progeny differed in their ability to survive after being transferred to a new host.

When reanalyzing the raw data of PJM1 nanopore (ONT) long-reads, no reads covering the decayed MRCP were found. We speculate that the inherently low excision frequency of MRCP along with multiple excision types helped in reducing the content of residual reads below a detectable threshold. Meanwhile, we also noticed that the gene *siiE* truncated by the MRCP on the chromosome is a large open reading frame (16-kb), which encodes a highly repetitive giant protein SiiE (5,559 aa) composed of 53 Ig domains (Barlag and Hensel, 2015). Such a long, repetitive sequence may have also played a part in hindering the detection of the degradation structure of MRCP in chromosome.

There is scarce up-to-date literature regarding Hfr strains, especially concerning multi-resistant Hfr strains. However, the ability of Hfr strains to spread ARGs should not be ignored. In this regard, strain SHfr_HI2_^R^ was able to generate free IncHI2 plasmids featuring the same size as the chromosomal integration state or generate plasmid featuring smaller sizes. Besides, the presence of R_HI2_’ factors and of the chromosomal integrated state was observed in the *E. coli* receptor, albeit in high or low frequencies, respectively. Notably, although MRCP was integrated into the chromosome of recipient *E. coli* J53, the “pseudo-Hfr” transconjugant was not transferrable to other bacterial hosts. Hence, *E. coli* strains presenting the chromosomal integrated state (i.e., PJ-T13) should not be regarded as Hfr strain in spite of harboring mating-related genes. Taken together, the data pertaining to the strain SHfr_HI2_^R^ reported in this study further expands the scope of Hfr strains.

## Conclusion

The emergence of polymorphic MGEs in a given strain caused by MRCP, may accelerate the bacterial response to adverse environments. The MRCP possesses conjugative traits but endows the host cell with a biphasic lifestyle, which ensured MRCP maintained a semi-equilibrium state in PJM1, increasing its persistence in the host cell. In this regard, PJM1 could act as plasmid reservoir and transfer ARGs to other strains continuously. The relationship between the mechanisms of lateral and vertical transmission of bacterial resistance may be more complex than previously perceived. Understanding the role and clinical significance of MGEs in the spread of antibiotic resistance is essential to prevent the global spread of AMR.

## Data Availability Statement

The datasets presented in this study can be found in online repositories. The names of the repository/repositories and accession number(s) can be found in the article/Supplementary Material.

## Author Contributions

M-XC and H-XJ conceived and designed the experiments. M-XC, JNZ, X-MD, JEZ, and YL performed the experiments. M-XC and J-FZ analyzed the data and drafted the manuscript. M-XC, RL, and H-XJ revised the manuscript. All authors approved the final draft and contributed to the article and approved the submitted version.

## Conflict of Interest

The authors declare that the research was conducted in the absence of any commercial or financial relationships that could be construed as a potential conflict of interest.

## Publisher’s Note

All claims expressed in this article are solely those of the authors and do not necessarily represent those of their affiliated organizations, or those of the publisher, the editors and the reviewers. Any product that may be evaluated in this article, or claim that may be made by its manufacturer, is not guaranteed or endorsed by the publisher.
