# Supplementary material for: Formation, Transmission, and Dynamic Evolution of a Multidrug-Resistant Chromosomally Integrated Plasmid in Salmonella Spp
Source: Front Microbiol. 2022 Apr 6;13:846954. doi: 10.3389/fmicb.2022.846954 (PMC9019673; doi:10.3389/fmicb.2022.846954)
Supplement: Supplementary file 1 [file Data_Sheet_1.PDF]

Supplementary data

Table S1 Primers used in this study.

Primer ID	Sequence (5'-3')	Primer location	Target	Size (bp)	references
bla$_{CTX-M-1G}$-F	ATCCCATGGTTAAAAAATCACTGC	*bla*$_{CTX-M-55}$	*bla*$_{CTX-M-55}$	890	(Zhang et al., 2019)
bla$_{CTX-M-1G}$-R	CCGTTTCCGCTATTACAAACCGTTG				
qnrS-F	ACGACATTCGTCAACTGCAA	*qnrS*	*qnrS*	417	(Zhang et al., 2019)
qnrS-R	TAAATTGGCACCCTGTAGGC	*qnrS*			
HI2-F	TTTCTCCTGAGTCACCTGTTAACAC	*iterons*	*iterons*	644	(Zhang et al., 2019)
HI2-R	GGCTCACTACCGTTGTCATCCT	*iterons*			
E-F	TTGAACGTCCATTCGCCCTTGT	3'-Δ*siiE*	Normal *siiE* or residual segments after excision of MRCP from the chromosome of PJM1	> 5000, 2255 or 1487	This study
E-R	TTAAAAAAGCAGCTTGACGAGGCTGA	5'-*siiE*Δ			
F-F	CTGTTGCTGTCCGGATGACAGG	*siiF*	*siiF*	405	This study
F-R	CAGTATGACGCTCAATCTCCGAT				

Table S2 Copies of different insertion sequences in pOYZ4 and MRCP

	IS*1R*	IS*Kpn18*	IS*150*	IS*Kpn26*	IS*102*	IS*26-v1*	IS*26*	IS*100kyp*	IS*Ec78*	IS*Kpn19*	IS*Ecp1*	IS*4321R*	IS*Vsa3*
MRCP	1	1	1	1	1	4	6	1	1	1	1	0	0
pOYZ4	1	0	1	1	1	3	7	0	1	1	1	1	1

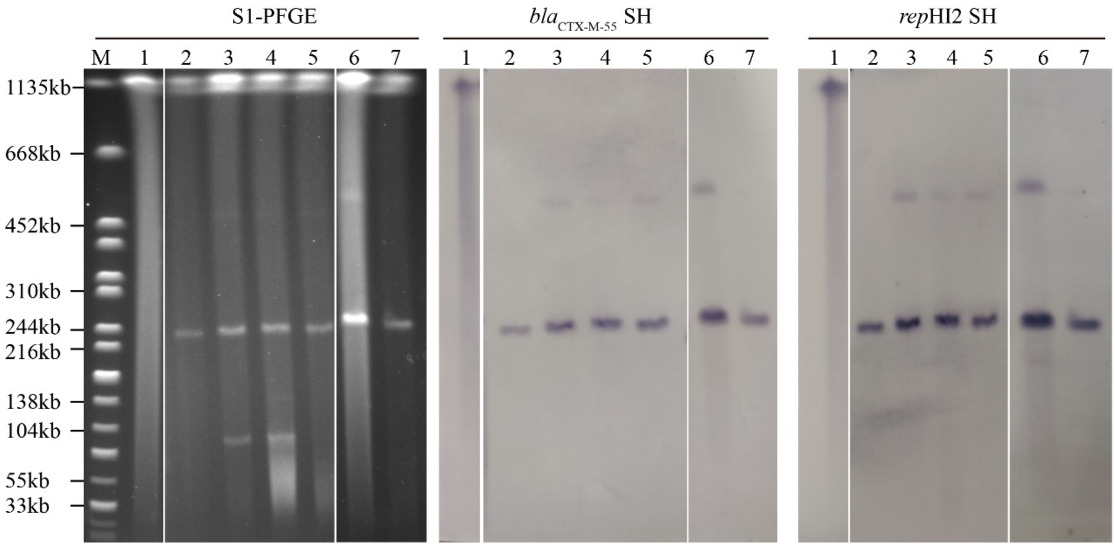

FIG S1 S1-PFGE and Southern hybridization with *bla*$_{CTX-M-55}$ and *rep*HI2 probe. Lane 1, PJM1; lane 2, PJ-T0, transconjugant from PJM1 to J53; lane 3, PJ-T0-LT, transconjugant from PJ-T0 to str.14028s; lane 4, PJ-T0-SL, transconjugant from PJ-T0 to SL1344; lane 5, PJ-T0-M39 transconjugant from PJ-T0 to M39; lane 6, OYZ4; lane 7, C-OYZ4; M, H9812 as a marker. SH, Southern hybridization using the *bla*$_{CTX-M-55}$ or *rep*HI2 probes. Transfer from other J53 transconjugants (PJ-T1, PJ-T7, PJ-T15, PJ-T16 and PJ-T17) are similar to that from PJ-T0, and the results are not shown.

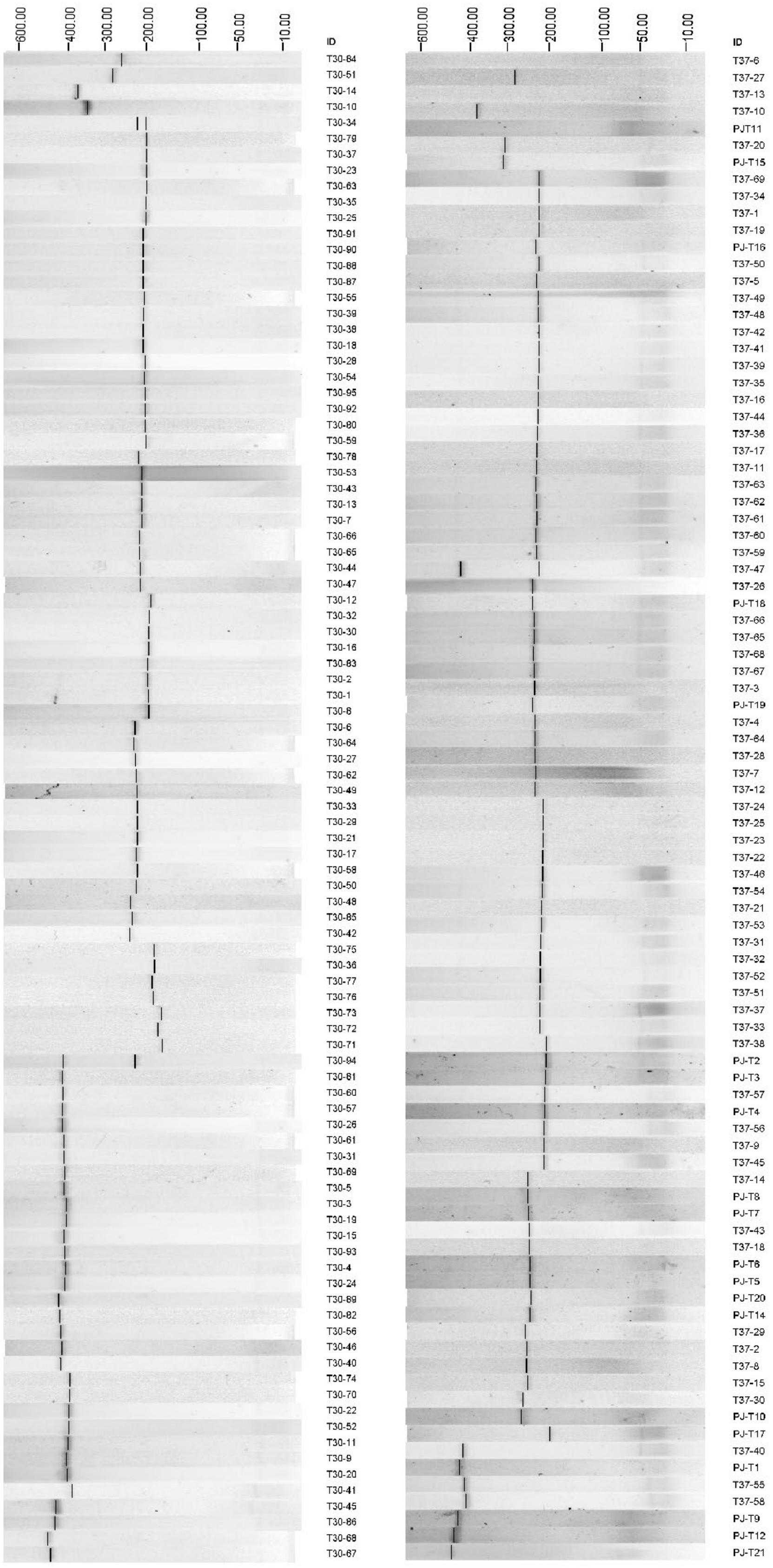

FIG S2 S1-PFGE of transconjugants containing MRCP progenies from PJM1. The left and right panels were the transconjugants obtained at 30°C and 37°C, respectively.

a.

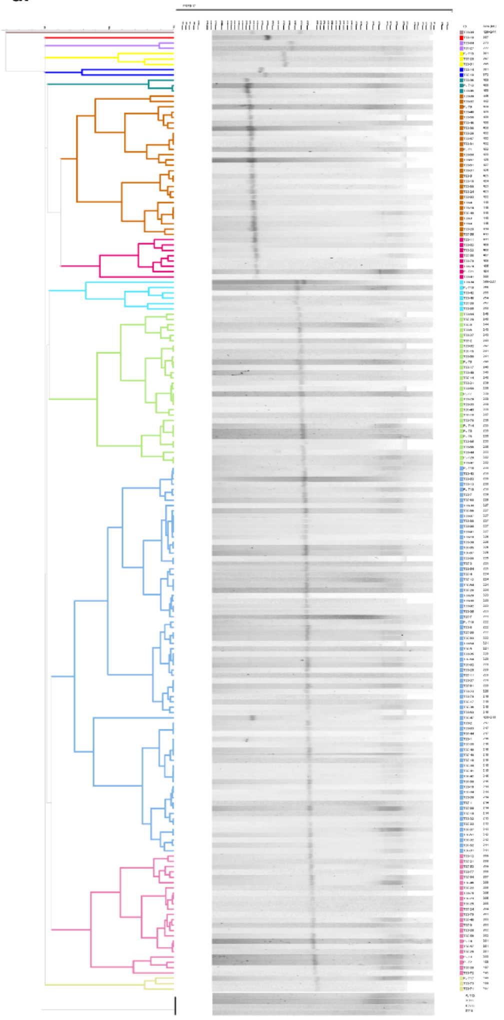

Clusters	Plasmid size (kb)	Num & Rate (%) 30 °C	37 °C	Sum
A	426+244	1 (1.1)	0 (0)	1 (0.5)
	357	1 (1.1)	0 (0)	1 (0.5)
	272~273	1 (1.1)	1 (1.1)	2 (1)
	295~301	1 (1.1)	2 (2.2)	3 (1.6)
	372~381	1 (1.1)	1 (1.1)	2 (1)
	456~459	2 (2.1)	1 (1.1)	3 (1.6)
	413~446	23 (24.2)	4 (4.4)	27 (14.6)
	398~411	6 (6.3)	2 (2.2)	8 (4.3)
B	250~256	4 (4.2)	2 (2.2)	6 (3.2)
	232~246	16 (16.8)	13 (14.4)	29 (15.7)
	211~230	31 (32.6)	42 (46.7)	73 (39.5)
	195~209	6 (6.3)	17 (18.9)	23 (12.4)
	187~190	2 (2.1)	1 (1.1)	3 (1.6)
C	Chr	0 (0)	4 (4.4)	4 (1.6)
	Total	95	90	185

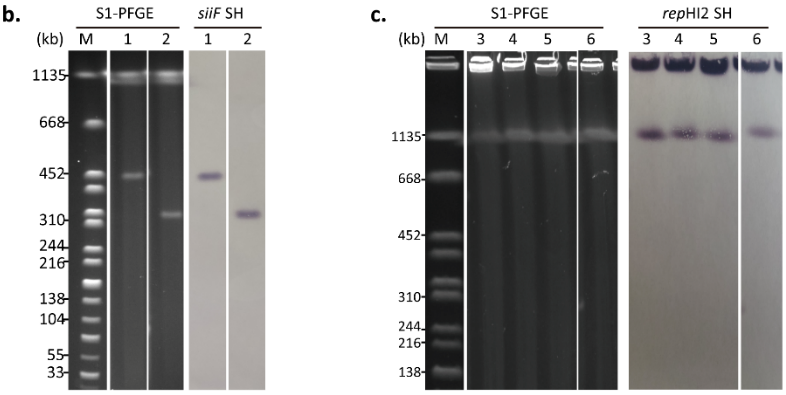

FIG S3 S1-PFGE and Southern hybridization of transconjugants containing MRCP progenies from PJM1. (a) Dendrogram obtained by cluster analysis of the S1-PFGE profiles of 185 *E. coli* J53 transconjugants, 95 of which were obtained at 30°C, and 90 were obtained at 37°C. S1-PFGE and Southern hybridization (b) with the *siiF* probe for transconjugants carrying plasmids larger than 260 kb and (c) *rep*HI2 probe for the four transconjugants in cluster C, respectively. Lane 1, PJ-T1, transconjugant harbored the 420 kb plasmid; lane 2, PJ-T15, transconjugant harbored the 320 kb plasmid; lane 3~6 the four transconjugants in cluster C. M, H9812 as a marker. SH, Southern hybridization.

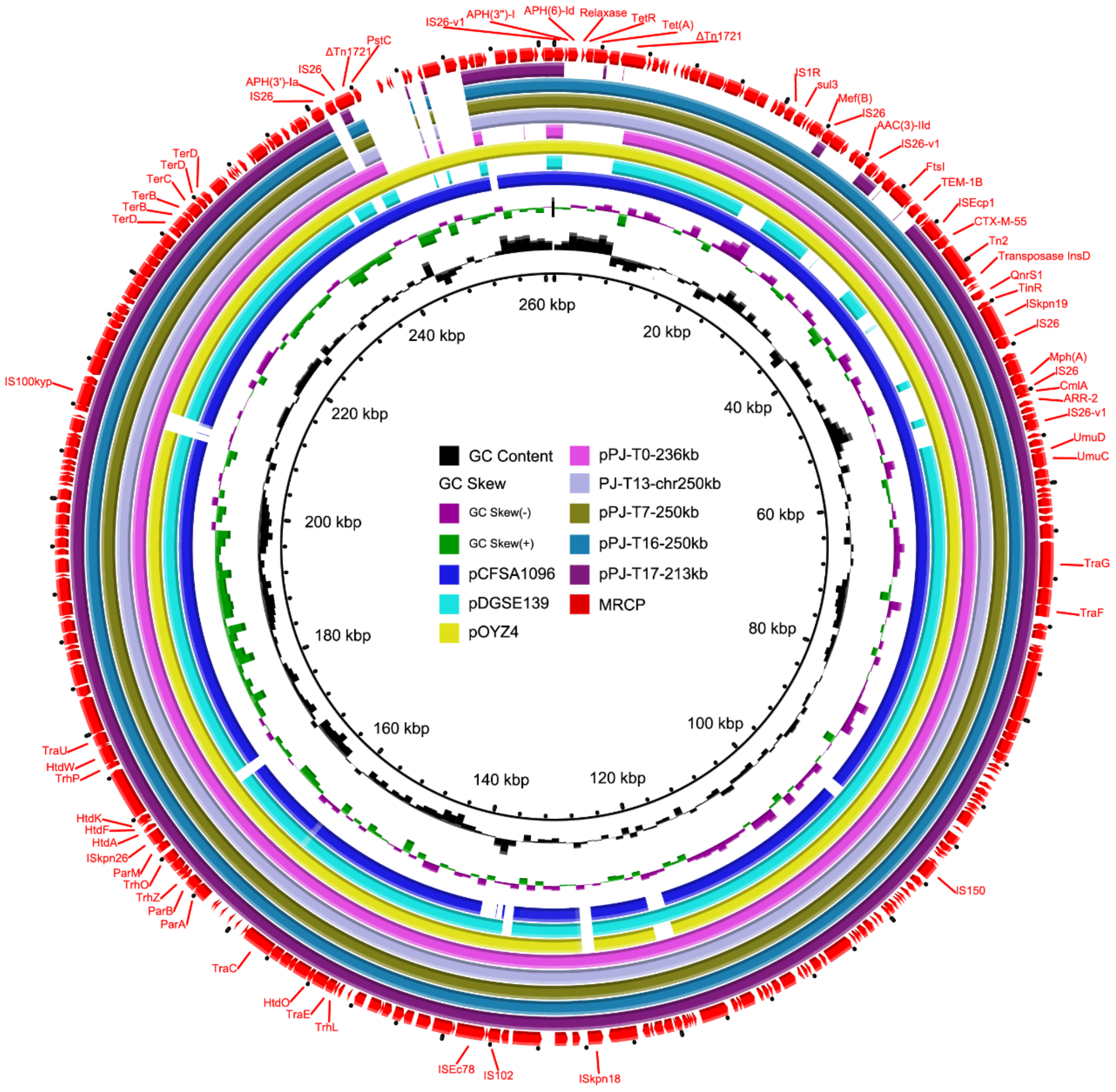

FIG S4 Circular alignment of MRCP and its progeny with other similar IncHI2 plasmids. Outermost red circle denotes MRCP as reference. basic genetic loci in MRCP are tagged. Deep purple, gray bule, olive-green, lilac and violet circles represent pPJ-T17-213kb, pPJ-T16-250kb, pPJ-T7-250kb, PJ-T13-chr250kb and pPJ-T0-236kb, respectively; the yellow circle represents pOYZ4; the aqua and bright blue circles represent the plasmids obtained from GenBank, pDGSE139 (KM198330) and pCFSA1096 (CP033347.1).

References

Zhang, C.Z., Ding, X.M., Lin, X.L., Sun, R.Y., Lu, Y.W., Cai, R.M., et al. (2019). The Emergence of Chromosomally Located bla CTX-M-55 in Salmonella From Foodborne Animals in China. *Front Microbiol* 10**,** 1268. doi: 10.3389/fmicb.2019.01268.
